# Isolation, identification, and production optimization of natural functional pigments produced by *Talaromyces atroroseus* LWT-1

**DOI:** 10.3389/fmicb.2025.1612109

**Published:** 2025-06-18

**Authors:** Xian Xia, Li-Yu Liu, Miao Liu, Guo-Jun Hu, Wen-Ting Li, Zi-Yi Wang, Yao Pei, Yan-He Li, Jing-Jing Li, Yan-Xiang Wang, Xiao-Shan Shi, Jun-Ming Tu

**Affiliations:** ^1^Hubei Key Laboratory of Edible Wild Plants Conservation and Utilization, Huangshi Key Laboratory of Lake Environmental Protection and Sustainable Utilization of Resources, Hubei Engineering Research Center of Characteristic Wild Vegetable Breeding and Comprehensive Utilization Technology, Hubei Normal University, Huangshi, China; ^2^Hubei Key Laboratory of Natural Medicinal Chemistry and Resource Evaluation, School of Pharmacy, Tongji Medical College, Huazhong University of Science and Technology, Wuhan, China; ^3^Laboratory for Functional Foods and Human Health, Center for Excellence in Post-Harvest Technologies, North Carolina Agricultural and Technical State University, Kannapolis, NC, United States

**Keywords:** natural pigments, *Talaromyces atroroseus*, purification, cytotoxicity assay, production optimization

## Abstract

Natural microbial pigments are gaining attention for their potential in various applications due to their safety and sustainability. In this study, we isolated a high-yielding pigment-producing fungus identified as *Talaromyces atroroseus* LWT-1. The predominant pigment compounds were isolated from the culture extract of *T. atroroseus* LWT-1 through various chromatographic methods and identified as talaroconvolutins A (**1**) and B (**2**), and talarofuranone (**3**). Compounds **1** and **3** exhibited cytotoxic activity against MCF7, Huh-7, and H446 lines with IC_50_ values ranging from 0.68 ± 0.09 to 4.19 ± 0.71 μM. In contrast, compound **1** was non-toxic to BEAS-2B cells and significantly promoted their proliferation. To optimize pigments yield, we conducted a series of single-factor and orthogonal experiments to determine the optimal fermentation conditions. The optimal conditions were determined to be: SDA culture medium, 32°C incubation temperature, 170 rpm shaking speed, 60 mL working volume in a 250 mL flask, and a culture duration of 120 h.

## 1 Introduction

Although artificial pigments initially replaced the natural ones (Aman Mohammadi et al., 2022), they are mostly non-metabolizable, lack nutritional value, and, in some cases, present potential health hazards. Additionally, the production of synthetic pigments is associated with high energy consumption and significant waste emissions, posing risks to both human health and the environment. In response, many countries have implemented regulations restricting the use of harmful synthetic pigments to mitigate their associated risks (Mirjalili et al., 2011). This shift has driven researchers to seek alternative pigment resources, bringing natural pigments, for their safety and potential health benefits, back into focus. As a result, interest in natural pigments has surged, encouraging their expanded use (Mohammad Azmin et al., 2022).

Natural pigments can be broadly classified into three categories: animal-, plant-, and microbe-derived pigments (Fernández-López et al., 2020; Liu et al., 2022; Maoka, 2020; Parichy, 2009; Zhu et al., 2023). As the demand for natural pigments continues to grow, the limited availability of animal- and plant-derived pigments has shifted attention toward pigment-producing microorganisms. Compared to animal and plant pigments, microbial pigments offer several advantages, including diverse chemical structures, potent biological activity, abundant microbial resources, ease of extraction, and suitability for large-scale fermentation. Furthermore, their production is environmentally friendly and does not lead to species depletion. In summary, microbial pigments represent a more sustainable and scalable alternative to traditional pigment sources (Muhammad et al., 2024; Wang et al., 2017). The first successfully microbial pigment developed for use in food is beta-carotene, produced by the fungus *Blakeslea*, which was approved as a food ingredient by the European Union in 1995 (Dufossé et al., 2005). Other microbial pigment have found applications across various industries. Notable examples include bacterioruberin, tyrian purple, and melanin derived from *Aspergillus niger* (Koch et al., 2023; Mussagy et al., 2024; Lee et al., 2021). Monascus pigment has been widely used in Southeast Asian areas, particularly in China, Japan and Indonesia (Long et al., 2023). However, it has been banned in certain regions, most notably in the European Union, where its consumption is strictly prohibited. The ban is primarily due to scientific uncertainty regarding its potential health risks. Although research on microbial pigment production is still in its early stages, there is growing interest in environmentally friendly alternatives to synthetic dyes. Microorganisms have gained considerable attention due to their ability to produce natural pigments on an industrial scale at relatively low cost (Rather et al., 2023).

In this study, we isolated a pigment-producing fungus, *Talaromyces atroroseus* LWT-1, which does not produce any known mycotoxins and is considered safe for use in food and cosmetic applications (Frisvad et al., 2013). The major pigment was isolated and purified with various chromatographic techniques and its structure was elucidated as talaroconvolutin A by spectroscopic analysis. Talaroconvolutin A exhibited notable antitumor activity and good selectivity, indicating its potential as a functional food pigment. To enhance pigments yield, we optimized the culture medium and fermentation parameters through single-factor and orthogonal design experiments.

## 2 Materials and methods

### 2.1 Microorganism and media

*Talaromyces atroroseus* LWT-1 was isolated from the laboratory environment and stored at −80°C in 20% (w/v) glycerol stock solution. The strain was deposited in China Center for Type Culture Collection (Wuhan University, Wuhan, China) with CCTCC NO M2024681.

The culture media (PDA, CA, CZ, YPD, YEA, SDA, MEA, and CYA) used in the subsequent experimental steps were presented in the Supplementary material. A small amount of mycelium was then collected to prepare mounts, which were examined under a light microscope (AM200AD Advanced Microscope and Computerized Image Acquisition System, Suzhou Aseet Optical Instrument Co., China) to observe the morphological characteristics of the mycelium. Total DNA was extracted using the CTAB (cetyltrimethylammonium bromide) method for molecular characterization and amplified by PCR with primers ITS1 (TCCGTAGGTG AACCTGCGG) and ITS4 (TCCTCCGCTTATTGATATGC). The PCR products were purified using an Axygen DNA Gel Recovery Kit (Corning Bioscience Co., Ltd., China), and sequencing was performed by Beijing Kengke Biotechnology Co., Ltd.^1^ The resulting ITS sequences were subjected to BLAST in NCBI.^2^ Sequences with higher homology were selected for genetic distances calculation using MEGA11.0 software, employing the Kimura 2-parameter method. The neighbor-joining (NJ) method was used to construct a phylogenetic tree and assess the species relationship of strain.

### 2.2 Materials

The device, instruments, and reagents used in the present work are same as that used in our previous reports (Shi et al., 2019).

### 2.3 Fermentation, extraction, isolation, and structural identification

The fresh mycelia of *T. atroroseus* LWT-1 was inoculated into 2 L flask preloaded with 1 L of SDA medium followed by a five-day culture at 150 rpm/min and 28°C. The whole liquid (1,000 mL) was transferred as seed into a 100 L fermentor preloaded with 50 L of sterilized SDA medium and cultivated at 28°C for 1 month. The whole fermented cultures were extracted with EtOAc for three times, and the solvent was evaporated under reduced pressure to afford an organic extract (109.3 g). The extract was fractionated by Si gel vacuum liquid chromatography (VLC) using different solvents of increasing polarity from petroleum ether (PE) to MeOH to yield 10 fractions (Frs. 1–10) based on TLC and HPLC analysis. Purification of Fr. 5 (11.9 g) by reversed-phase column chromatography (CC) over Lobar LiChroprep RP-18 with a MeOH-H_2_O gradient (from 10: 90 to 100: 0) yielded 10 subfractions (Frs. 5.1–5.10). Fr. 5.8 (7.3 g) (MeOH-H_2_O gradient from 70: 30 to 90: 10) was purified by CC on Si gel eluting with a CH_2_Cl_2_- EtOAc gradient (from 400: 1 to 2: 1) to obtain 3 fractions (Fr. 5.8.1–5.8.3). Fr. 5.8.1 by pTLC (developing solvents: PE-EtOAc, 1: 1) and CC on Sephadex LH-20 (MeOH) to obtain compound **1** (5.8 g). Fr. 5.8.2 by pTLC (developing solvents: PE- Acetone, 5: 1) and CC on Sephadex LH-20 (MeOH) to obtain compound **2** (15.3 mg). Fr. 5.8.3 by pTLC (developing solvents: PE- Acetone, 3: 1) and CC on Sephadex LH-20 (MeOH) to obtain compound **3** (14.2 mg).

The purified pigments were dissolved in deuterated chloroform, and transferred into a clean NMR tube for NMR analysis (NMR instrument, Bruker, Germany).

### 2.4 Cytotoxicity assay

The cytotoxic activities of the isolated pigments against the normal cell lines BEAS-2B (human normal lung bronchial epithelial cells) and the three tumor cell lines H446 (human lung cancer cell line), MCF2 (human breast cancer cell line), and Huh-7 (human hepatocarcinoma cell line) were determined according to previously reported methods (Zhang et al., 2024). Briefly, H446, MCF2, Huh-7, and BEAS-2B cells were counted and resuspended. The cell suspension was inoculated into a 96-well plate with 5 × 10^3^ cells per well. The cells were treated with different concentrations of the isolated pigments for 48 h. Then, the cell viability was determined with CCK8 kits at 450 nm using a Multiskan GO microplate reader (ThermoFisher Scientific). The half maximal inhibitory concentration (IC_50_) values of the isolated pigments were calculated by Graphpad Prism and the cell viability of compound **1** was calculated using the formula: cell viability = [(A_s_-A_b_)/(A_c_-A_b_)] × 100%. In this formula, where As is the absorbance of the test group. Ab is the blank, and Ac is the control. Cisplatin was used as positive control. The experiment was repeated three times.

### 2.5 Production optimization of the pigments produced by LWT-1

#### 2.5.1 Effects of the different culture media

*Talaromyces atroroseus* LWT-1 was inoculated onto eight different solid media: CA, CZ, YPD, YEA, SDA, MEA, and CYA, with three replicates for each treatment. The inoculated plates were incubated at 28°C in a constant temperature incubator. The growth of the colonies on different media was measured by the crosshatch method at various time points (2, 4, 6, 8, 10, and 12 days), and photographs were recorded their growth. On the 12th day of cultivation, the medium was cut into pieces by using Tween 80 to treat the surface mycelium. Pigments were extracted using EtOAc (w: v = 1: 2) as a solvent at 28°C, with a rotational speed of 170 rpm, and the continued for 24 h (Bouhri et al., 2020). Then, the extraction was filtered under reduced pressure, and the absorbance was measured at the optimal wavelength using a UV spectrophotometer (SPECORD plus200 UV–Vis Spectrophotometer, analytikJena, Germany).

#### 2.5.2 Effects of fermentation conditions

The effects of incubation temperature (18°C, 20°C, 24°C, 28°C, and 32°C), shaker volume (40 mL, 60 mL, 80 mL, 100 mL, 120 mL, and 140 mL per 250 mL flask), shaker speed (110 r/min, 130 r/min, 150 r/min, 170 r/min, and 190 r/min), inoculum size (1, 3, 5, 7, 9, and 11% v/v), and incubation time (data collected every 24 h over 10 days) on fungal fermentation were systematically studied. The control group was incubated with a 40% medium loading factor at 28°C, 150 r/min for 5 days, using a 1% (v/v) inoculum. Each condition was conducted in triplicate to ensure reproducibility.

#### 2.5.3 Orthogonal experiments on optimum incubation temperature, shaker volume, shaker speed, and incubation time

Based on the results of the one-factor *experiments*, the next step involved the comprehensive selection of incubation temperature, shaker speed, shaker filling volume, and incubation time. To optimize these factors, an orthogonal test of L_9_(3^4^) was designed using an orthogonal assistants tool. The factor levels for the orthogonal *experiments* are shown in Supplementary material.

### 2.6 Determination of the color value

The mycelium cultured liquid was filtered using 200-mesh gauze, and the filtrate was appropriately diluted. The absorbance of the diluted solution was measured using 1 cm optical cuvette at the optimum wavelength, with distilled water as a blank control. The filtrate color value (U/mL) was calculated by multiplying the absorbance value by the dilution factor.

### 2.7 Statistical analysis

All statistical analyses were conducted using GraphPad Prism5 (GraphPad Software, La Jolla, California) and expressed as the mean ± SEM. Comparisons among three or more groups were performed using one-way analysis of variance. *p* < 0.05 was considered statistically significant.

## 3 Results

### 3.1 Isolation and identification of strain LWT-1

**Figure 1:**
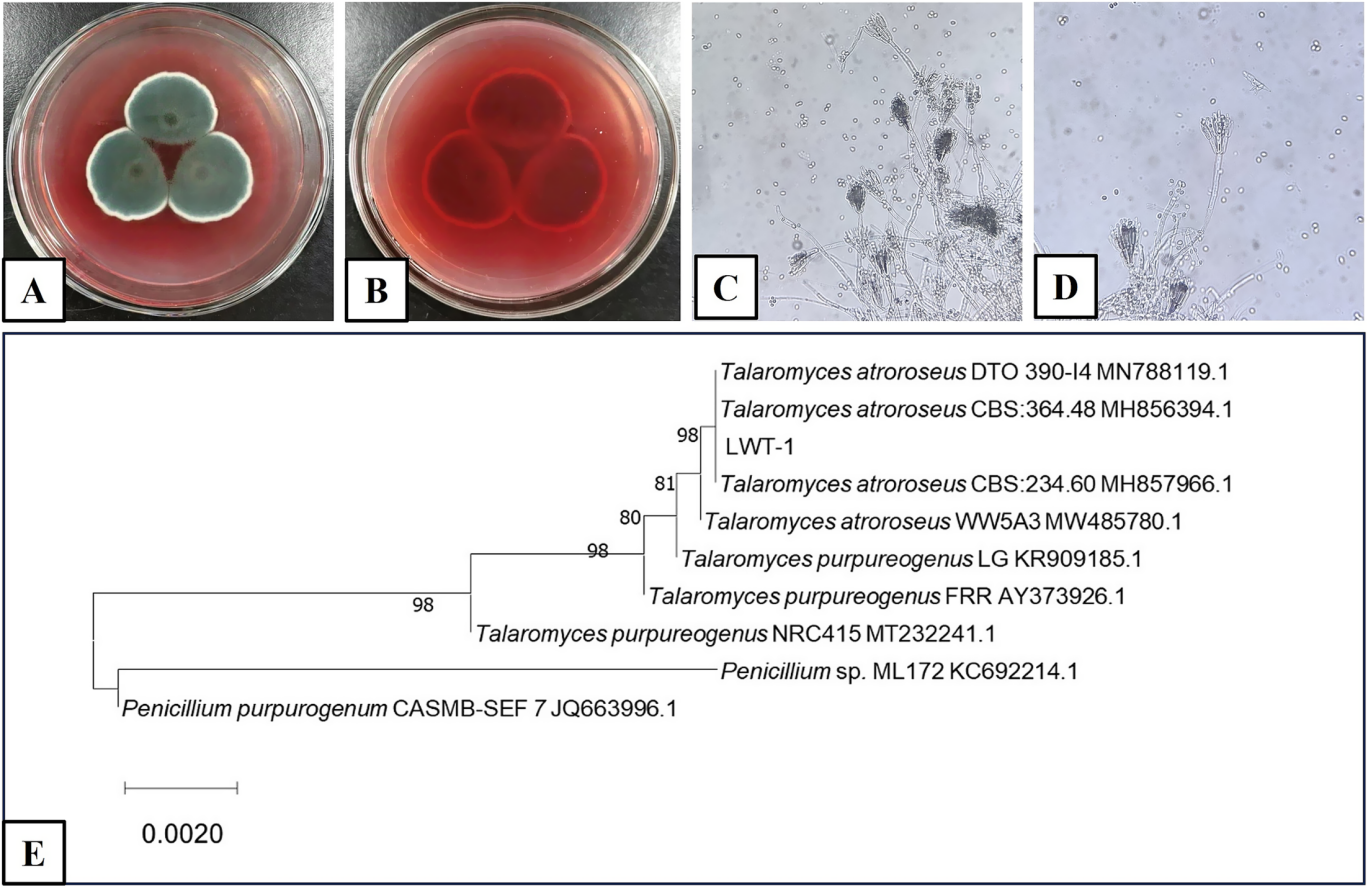
Morphology of *T. atroroseus* LWT-1 **(A–D)** and ITS phylogenetic tree constructed using the NJ method **(E)**. **(A,B)** Colonies on SDA medium (**A**–obverse view and **B**–reverse view); **(C)** Mycelium; **(D)** Conidiophores.

The fungus was analyzed through both classical morphological and molecular methods. Morphologically (Figures 1A–D), on SDA at 25°C, colonies exhibited a floccose and velvety texture; mycelia were white and olive green; sporulation was strong; soluble pigments were red; and the reverse side of the colonies was dark red. The mycelium appeared dark green with transverse septa, and the conidia (which are asexual spores in the context of *Penicillium* and *Talaromyces,* as indicated by their production without sexual fusion and direct formation from hyphae) were ellipsoidal with broom-like branched peduncles, suggesting it belonged to *Penicillium* sp. based on traditional morphological criteria often associated with asexual reproductive structures in these genera.

Molecularly (Figure 1E), ITS sequence comparison and phylogenetic tree construction indicated a high degree of homology with *T. atroroseus*. While *T. atroroseus* is also capable of sexual reproduction (producing sexual spores, specifically ascospores, under appropriate conditions), the morphological characteristics observed here are indicative of its asexual phase. Based on the combined morphological evidence of asexual conidiation and molecular phylogenetic analysis, the fungus was identified as *T. atroroseus* LWT-1. This identification aligns with the understanding that *Talaromyces* species, including *T. atroroseus*, can exhibit both asexual (via conidia) and sexual (via ascospores) reproductive strategies, though the asexual phase is often more readily observed in laboratory settings (Yilmaz et al., 2014).

### 3.2 Isolation, purification, and structural characterization of pigments

**Figure 2:**
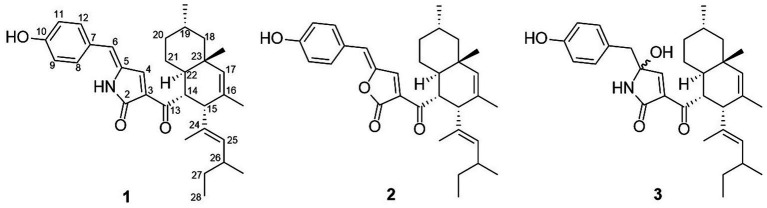
Chemical structures of pigment compounds **1**–**3**.

The EtOAc extracts of *T. atroroseus* LWT-1 were chromatographed on normal- and reversed-phase silica gel, Sephadex LH-20, as well as prep. TLC to yield pigment compounds **1**–**3** (Figure 2). Their structures were identified as talaroconvolutins A (**1**) and B (**2**), and talarofuranone (**3**) by comparing their NMR data in the Supplementary material with those reported in the literature (Padmathilake et al., 2017; Suzuki et al., 2000).

**Figure 3:**
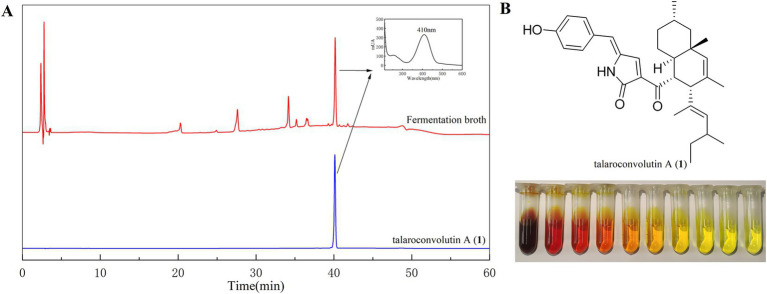
HPLC profiles of the fermentation broth and pure compound **1 (A)** and the color of compound **1** varies with its concentration **(B)**.

Compound **1** is a reddish pigment, appearing bright yellow when diluted (Figure 3B). Compound **2** is a red pigment, and compound **3** has a pale yellow hue. HPLC analysis of the fermentation broth and compound **1** revealed that compound **1** is the main pigment product and the maximum UV absorption wavelength of the pigment was 410 nm (Figure 3A). Therefore, 410 nm was selected as the optimal wavelength for determining the pigments color value in the fermentation broth.

### 3.3 Cytotoxicity

Compounds **1**–**3** were tested for their cytotoxicity against H446, Huh-7, and MCF7 cell lines. Compound **1** exhibited potent activity against all tested cell lines with IC_50_ values of 1.64 ± 0.14, 3.34 ± 0.47, and 4.19 ± 0.71 μM, respectively. Compound **3** exhibited cytotoxicity against ranging from 0.68 ± 0.09 to 3.74 ± 0.38 μM (Table 1). In contrast, compound **2** was inactive toward all tested cell lines. These data indicated that the tetramic acid five-membered ring instead of a furanone ring significantly increased the cytotoxicity of the isolated pigments (**1** and **3** vs. **2**).

**Table 1:** Cytotoxic inhibitory activities of compounds **1–3**.

Compounds	IC_50_ (μM)		
	H446^a^	Huh-7^b^	MCF7^c^
1	1.64 ± 0.14	3.34 ± 0.47	4.19 ± 0.71
2	>50	>50	>50
3	0.68 ± 0.09	1.60 ± 0.08	3.74 ± 0.38
DMSO	n.a.	n.a.	n.a.

a Human colorectal lung cancer cell line.

b Human hepatocarcinoma cell line.

c Human breast cancer cells line.

n.a.: no activity. Data shown are average ± SEM of 3 independent experiments.

**Figure 4:**
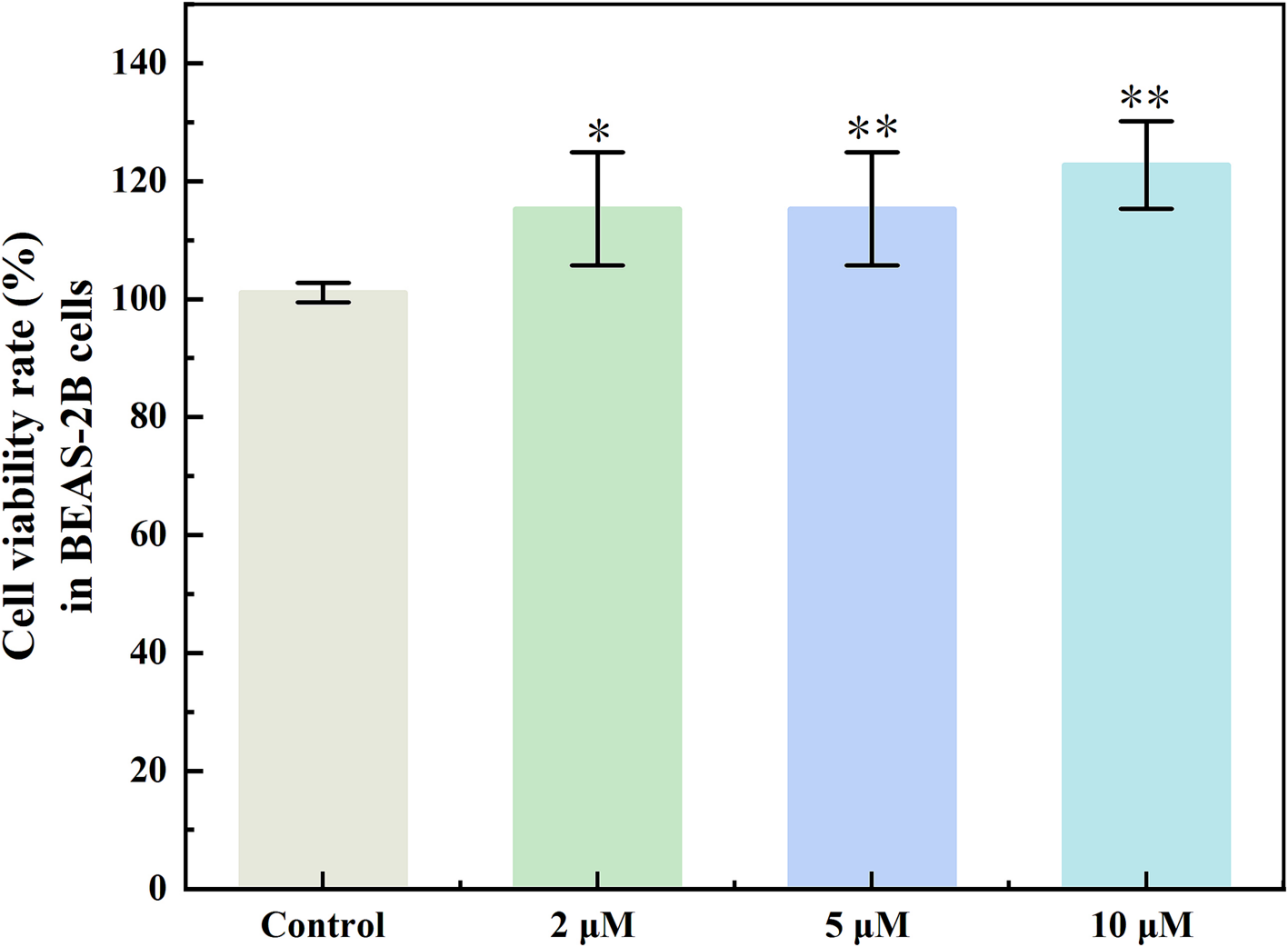
BEAS-2B cell survival under different concentrations of compound **1**. *p ≤ 0.05, **p ≤ 0.01.

Compound **1**, which was obtained in larger amounts, was further assayed for cytotoxicity tests to evaluate its effects on normal cells. The results indicated that compound **1** did not exhibit cytotoxicity toward **BEAS-2B** cells. Instead, an increased concentration of compound **1** was associated with a more pronounced promotion of cell growth (Figure 4).

### 3.4 Effects of the different culture media

The final pigment production of the fungal colonies were observed days of 2, 4, 6, 8, 10, and 12. The colonies of *T. atroroseus* were cultured on various media (PDA, CA, CZ, YPD, YEA, SDA, MEA, and CYA) at a constant temperature of 28°C. The status and pigment production were examined under these conditions to assess the optimal growth and pigment synthesis.

**Figure 5:**
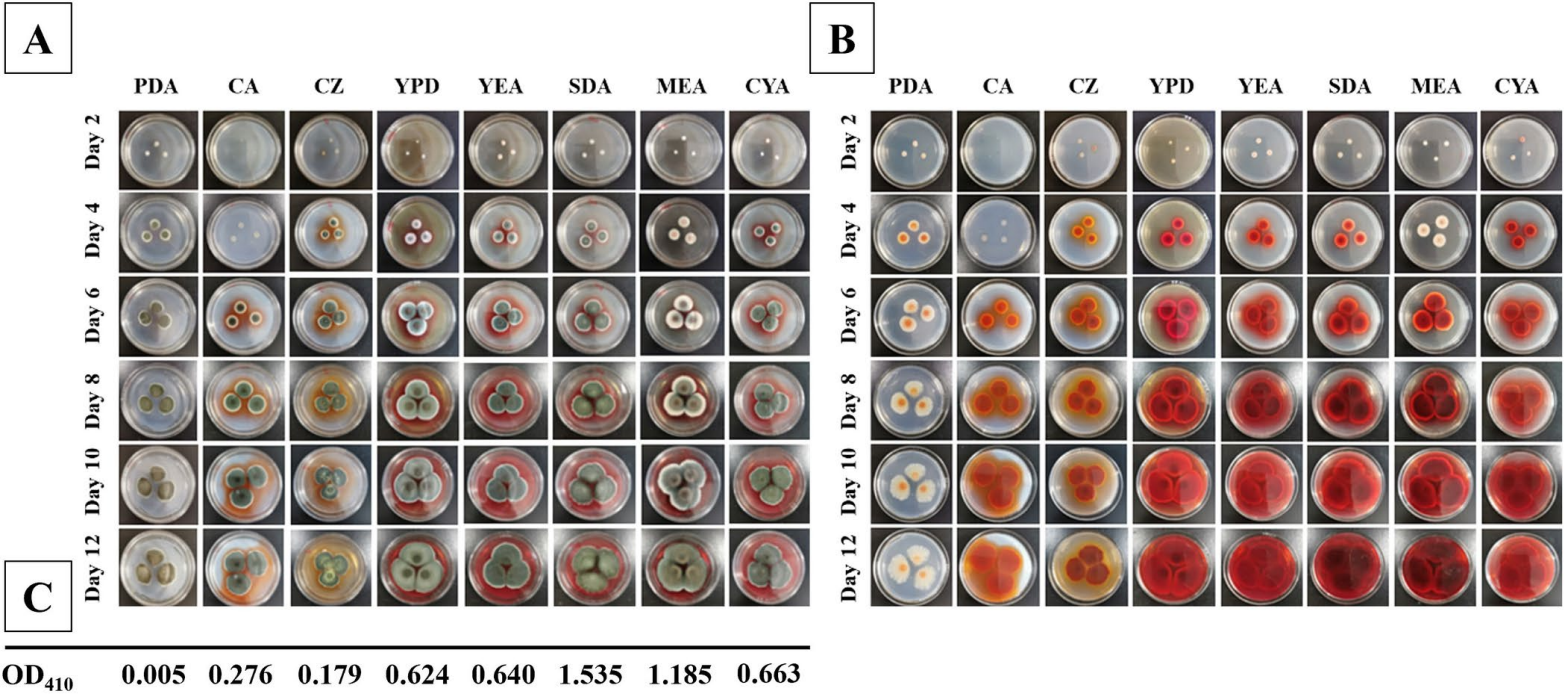
Colonies of *T. atroroseus* LWT-1 on different media (PDA, CA, CZ, YPD, YEA, SDA, MEA, and CYA) and different days of incubation (2, 4, 6, 8, 10, 12) (**A**−obverse view and **B**–reverse view) and the OD_410_ obtained after incubating each type of medium for 12 days **(C)**.

The fastest growth rate of *T. atroroseus* LWT-1 was observed on YPD, SDA, and MEA media, as shown in Figure 5. The colonies on SDA and MEA media produced the darkest pigment, a deep red color. After 12 days of growth, the pigment content was measured, with the highest absorbance value of 1.535 at OD_410nm_ observed in the SDA medium (Figure 5), indicating the highest pigment production. Therefore, SDA medium was determined to be the most suitable for the growth and pigment production of LWT-1 among the tested media.

### 3.5 Effects of fermentation conditions

**Figure 6:**
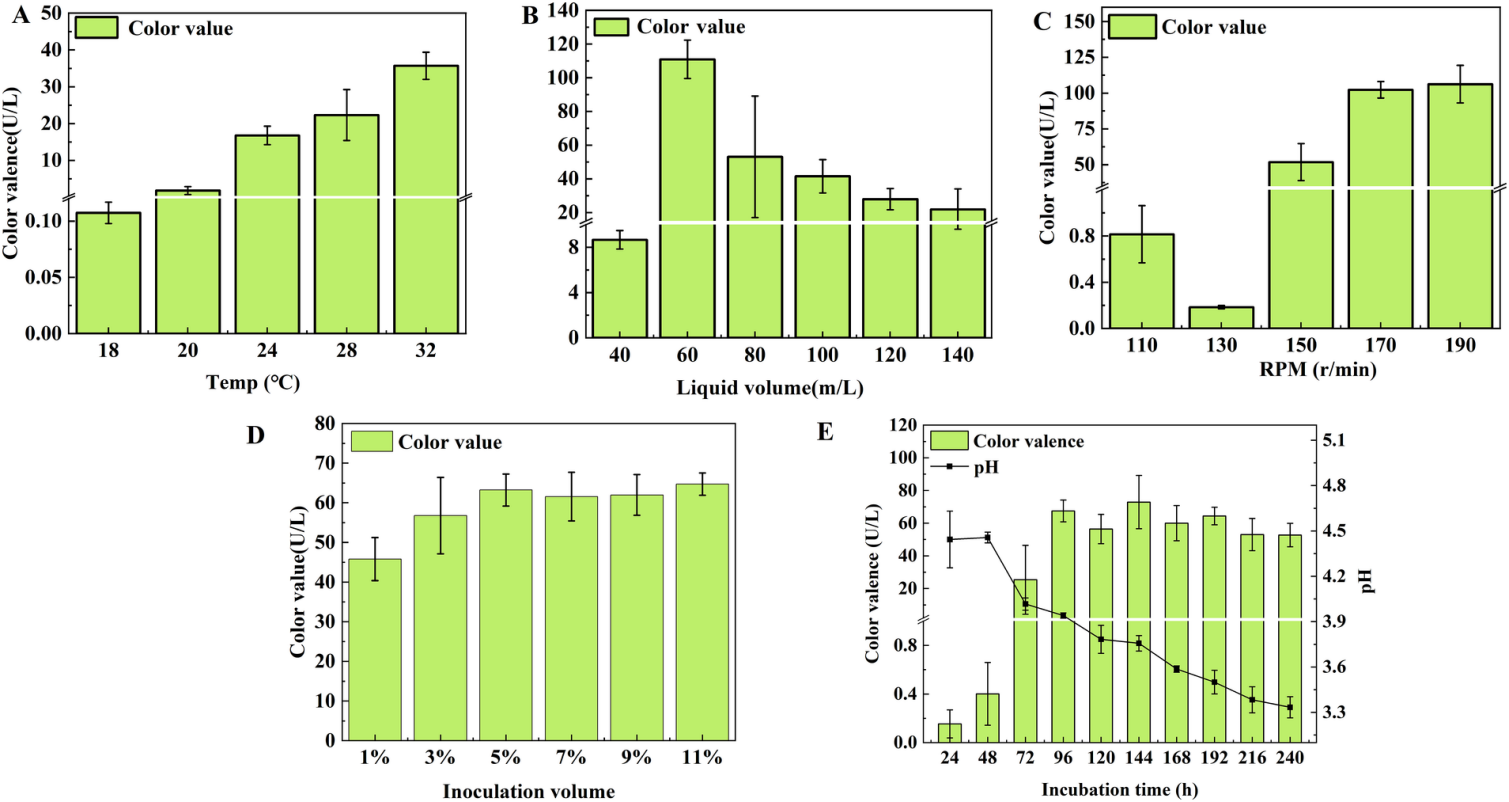
Effect of temperature **(A)**, liquid volume **(B)**, shaker speed **(C)**, inoculation volume **(D)**, and incubation time **(E)** on the color value of *T. atroroseus* LWT-1.

Strain LWT-1 was cultured under varying fermentation conditions, and the effects on pigment color value were analyzed. The color value increased with temperature, peaking at 36.011 U/mL at 32°C, which was significantly higher than at other temperatures (Figure 6A), making 32°C the optimal temperature. The highest pigment color value, 117.056 U/mL, was achieved with a 60 mL/250 mL shake flask filling volume, significantly higher than other conditions (Figure 6B). Therefore, a filling volume of 60 mL/250 mL was optimal for pigment production.

At shaker speeds, the color value reached 106.219 U/mL at 190 r/min and 102.293 U/mL at 170 r/min, but considering energy consumption, 170 r/min was the most suitable speed (Figure 6C). Regarding inoculum size, the highest color values were observed at 5 and 11% inoculum, with no significant difference between 3 and 11% (*P* ˃ 0.05), making 5% optimal (Figure 6D).

Pigment production increased from 24 to 96 h, reaching a maximum of 67.499 U/mL, with the pH dropping to 4.017 at 72 h. Since the pH range of 4–5 supported pigment accumulation, and further decreases led to mycelium autolysis, 96 h was the optimal incubation time (Figure 6E).

In conclusion, the optimal conditions for pigment production were a temperature of 32°C, a filling volume of 60 mL/250 mL, a rotational speed of 170 r/min, 5% (v/v) inoculum, and 96 h incubation time.

### 3.6 Orthogonal experiments of optimum incubation temperature, shaker speed, shaker volume, and incubation time

After a comprehensive analysis, temperature (**A**), rotational speed (**B**), loading volume (**C**), and incubation time (**D**) were selected as factors for the orthogonal tests. The results of the orthogonal tests were shown in Table 2, the analysis of extreme deviation indicated the relative influence of the four factors on pigment color value in the following order: incubation time (**D**) > loading volume (**C**) > temperature (**A**) > rotational speed (**B**). This analysis revealed that incubation time (**D**) had the most significant impact on the production of pigments by LWT-1.

**Table 2:** Results of orthogonal tests affecting the color value of pigments produced by LWT-1 (unit: U/mL).

Technique	Factor				Color value
	A	B	C	D	
1	1	1	1	1	28.565 ± 4.448
2	1	2	2	2	47.829 ± 15.203
3	1	3	3	3	32.530 ± 7.271
4	2	1	2	3	77.632 ± 5.417
5	2	2	3	1	21.163 ± 3.047
6	2	3	1	2	77.995 ± 1.454
7	3	1	3	2	42.624 ± 13.102
8	3	2	1	3	108.800 ± 3.136
9	3	3	2	1	41.963 ± 4.282
Average k_1_	39.644	49.607	71.787	30.564	
Average k_2_	58.93	59.264	55.808	56.149	
Average k_3_	64.462	54.166	35.442	76.324	
Range R	24.818	9.657	36.345	45.760	
Main factor	D>C>A>B				
Superior combination	A_3_B_2_C_1_D_3_				

The optimal combination of fermentation conditions identified through the orthogonal tests was A_3_B_2_C_1_D_3_. This corresponded to the following theoretical conditions for achieving the highest pigment color value: a temperature of 32°C, a rotational speed of 170 r/min, a loading volume of 60 mL, and an incubation time of 120 h. In conclusion, the orthogonal tests confirmed that the optimal fermentation conditions for maximizing the red pigment production of LWT-1 were A_3_B_2_C_1_D_3_, representing a robust framework for enhancing pigment yield under controlled conditions.

## 4 Discussion

Among various sources of natural pigments, microbial pigments are relatively easy to obtain and can be produced in large quantities through large-scale cultivation under appropriate conditions (Dufossé et al., 2005). In this study, we successfully isolated and characterized a high-yield pigment-producing fungal strain, *Talaromyces atroroseus* LWT-1. Its identity was confirmed using both morphological observation and molecular analysis. Notably, this strain demonstrated strong pigment-producing capacity without generating any known mycotoxins, highlighting its potential as a safe source of natural colorants for food and cosmetic applications (Frisvad et al., 2013). However, it is worth noting that previous studies on this strain primarily focused on its pigment-producing capability, without further investigating the chemical composition or the biological functions of the pigments beyond their coloring properties (Li et al., 2020). Furthermore, previous studies did not address the optimization of cultivation conditions, a critical step for enabling industrial-scale production. In contrast, our study filled this gap by systematically evaluating and optimizing fermentation parameters to enhance pigment yield. This study showed that SDA was the optimum medium for the pigment production. This might due to the high glucose content in SDA. Because glucose supports not only primary metabolic activities but also enhances the production of secondary metabolites, such as pigments (Huang et al., 2017). Moreover, the low pH of SDA can induce the expression of pigment-related genes in certain fungi. For example, *Cryptococcus neoformans* produces more melanin under acidic conditions (Baker and Casadevall, 2023). Additionally, the optimal combination of fermentation conditions identified through orthogonal testing was A_3_B_2_C_1_D_3_, corresponding to a temperature of 32°C, a rotational speed of 170 r/min, a loading volume of 60 mL, and an incubation time of 120 h. This combination yielded the highest theoretical color value of the red pigment, indicating that these parameters play a crucial role in pigment biosynthesis. Specifically, a temperature of 32°C may enhance the activity of metabolic enzymes, thereby promoting pigment production (Afshari et al., 2015). The moderate rotational speed of 170 r/min likely ensures adequate oxygen transfer, which supports both cell growth and the synthesis of secondary metabolites (Pfefferle et al., 2000). A loading volume of 60 mL appears to provide sufficient nutrients while maintaining a favorable gas–liquid ratio, and an incubation period of 120 h allows enough time for the accumulation of pigment (Bu et al., 2020). The synergistic effect of these fermentation parameters significantly promotes the increase in red pigment yield, providing reference data for future large-scale fermentation production.

Our results demonstrated that compounds **1** and **3** exhibited significant anticancer activity while also promoting the growth of normal cells. Notably, previous studies (Feng et al., 2020) did not assess the cytotoxicity of compound **1** on normal cells, an important aspect overlooked in earlier research. In contrast, our finding revealed this unique property of compound **1**, highlighting its potential as a functional food pigment with additional health benefits, particularly in cancer prevention supportive therapy. The cytotoxic activity exhibited by compounds **1** and **3**, but not compound **2**, against cancer cell lines underscores the critical role of chemical structure in modulating biological activity. Specifically, the presence of a tetramic acid five-membered ring in compounds **1** and **3**, rather than the furanone ring found in compound **2**, appears to be associated with enhanced cytotoxicity. This observation provides valuable insights into the structure–activity relationships of microbial pigments and may inform the future design of more potent anticancer agents. Previous research suggested that the selective cytotoxicity of compound **1** toward cancer cells may toward its interference with cell cycle regulation, induction of ferroptosis, and disruption of the MAPKs signaling pathway (Xia et al., 2020; Xia et al., 2023). Based on our findings, we hypothesize that the ability of compound **1** to promote normal cells growth may be attributed to more robust cell cycle regulation, enhanced antioxidant and stress response pathways, and potential non-specific effects in normal cells. However, these hypotheses require further experimental validation. While the total chemical synthesis of compound **1** has been reported (Yao et al., 2024), microbial fermentation offers a more viable strategy for industrial-scale production. It provides several advantages, such as a favorable safety profiles, reduced production costs, and precise control over molecular conformation—critical factors for ensuring both efficacy and scalability. Consequently, elucidating the biosynthetic pathway of compound **1** will likely become a central focus of future research efforts.

Investigation into the potential teratogenic and carcinogenic effects of synthetic dyes have raised significant concerns regarding their use in food products. Consequently, there is a urgent need to identify safe and natural alternatives to synthetic colorants (Aman Mohammadi et al., 2022). In recent years, a growing body of evidence has demonstrated that microbial pigments not only exhibit a wide range of biological activities but are also cost-effective to produce. For example, a green pigment with antibacterial and antiviral activities has been reported (Ibrahim et al., 2023), along with pyocyanin, which shows both antibacterial and antioxidant properties (Abdelaziz et al., 2023), and fungal pigment extracts that exhibit antitumor effects (El-Sayed et al., 2022). Our study corroborates and further expands upon these findings by identifying novel microbial pigments with demonstrated anticancer potential and favorable safety profiles.

## 5 Conclusion

In this study, the pigment-producing strain LWT-1 was identified as *Talaromyces atroroseus* based on morphological characteristics and molecular analysis. Talaroconvolutin A, a major pigment compound, was identified as a potent anticancer agent with selective cytotoxicity toward cancer cells while promoting the growth of normal cells under certain conditions. This dual functionality, selective cancer cell targeting alongside potential support for normal cell viability, highlights the therapeutic promise of microbial pigments. Optimal culture conditions were determined through one-way and orthogonal tests, using pigment yield in conjunction with mycelial biomass as evaluation criteria. The optimization of fermentation conditions provides a foundation for the industrial-scale production of microbial pigments, offering a sustainable alternative to synthetic dyes. Overall, these findings lay the foundation for the further development and application of *Talaromyces atroroseus* LWT-1-derived pigments. Future research should focus on further elucidating the underlying mechanisms of action, evaluating *in vivo* efficacy, and optimizing large-scale production to enable commercial viability.

## Data Availability

The datasets presented in this study can be found in online repositories. The names of the repository/repositories and accession number(s) can be found in the article/Supplementary material.

## Footnotes

^1^https://www.tsingke.com.cn/, Beijing, China.

^2^ https://blast.ncbi.nlm.nih.gov/Blast.cgi
