# Supplementary material for: Isolation, identification, and production optimization of natural functional pigments produced by Talaromyces atroroseus LWT-1
Source: Front Microbiol. 2025 Jun 18;16:1612109. doi: 10.3389/fmicb.2025.1612109 (PMC12213815; doi:10.3389/fmicb.2025.1612109)

Supplementary Material

Isolation, identification, and production optimization of natural functional pigments produced by *Talaromyces atroroseus* LWT-1

Xian Xia [1,2†], Li-Yu Liu [1†], Miao Liu [1], Guo-Jun Hu [1], Wen-Ting Li [1], Zi-Yi Wang [1], Yao Pei [1], Yan-He Li [3], Jing-Jing Li [1], Yan-Xiang Wang [1], Xiao-Shan Shi [1*], Jun-Ming Tu [1,*]

[1]Hubei Key Laboratory of Edible Wild Plants Conservation and Utilization, Huangshi Key Laboratory of Lake Environmental Protection and Sustainable Utilization of Resources, Hubei Engineering Research Center of Characteristic Wild Vegetable Breeding and Comprehensive Utilization Technology, Hubei Normal University, Huangshi, China,

[2]Hubei Key Laboratory of Natural Medicinal Chemistry and Resource Evaluation, School of Pharmacy, Tongji Medical College, Huazhong University of Science and Technology, Wuhan, China,

[3]Laboratory for Functional Foods and Human Health, Center for Excellence in Post-Harvest Technologies, North Carolina Agricultural and Technical State University, North Carolina Research Campus, 500 Laureate Way, Kannapolis, North Carolina, United States.

[†]These authors contributed equally to this work.

*Corresponding author: Xiao-Shan Shi (shixs@hbnu.edu.cn), Jun-Ming Tu (junming_tu@hbnu.edu.cn)

Table of Contents

Table S1. The culture medium used in the experiment and its constituent components;

Figure S1. ^{1}H NMR (600 MHz, $CDCl_3$) spectrum of compound **1**;

Figure S2. ^{13}C NMR (125 MHz, $CDCl_3$) and DEPT spectra of compound **1**;

Figure S3. COSY spectrum of compound **1**;

Figure S4. HSQC spectrum of compound **1**;

Figure S5. HMBC spectrum of compound **1**;

Figure S6. ^{1}H NMR (300 MHz, $CDCl_3$) spectrum of compound **2**;

Figure S7. ^{1}H NMR (300 MHz, $CDCl_3$) spectrum of compound **3**;

Table S1: The culture medium used in the experiment and its constituent components.

Culture media	Component and Content
PDA	potato 200g, glucose 20g, agar 15g, distilled water 1000 ml;
CA	sucrose 30g, K_2HPO_4 1g, $NaNO_3$ 3 g, KCl 0.5g, $MgSO_4$-$7H_2O$ 0.5g, $FeSO_4$-$7H_2O$ 0.01 g, agar 20 g, distilled water 1000mL;
CZ	sucrose 30g, $NaNO_3$ 2g, KCl 0.5g, $MgSO_4$-$7H_2O$ 0.5g, $Fe_2(SO_4)_3$ 0.01g, KH_2PO_4 0.65g, agar 20g, distilled water 1000mL;
YPD	tryptone 20g, glucose 20g, yeast extract 10g, agar 20g, distilled water 1000mL;
YEA	glucose 10g, yeast extract 5g, agar 20g, distilled water 1000mL
SDA	peptone 10g, glucose 40g, agar 20g, distilled water 1000mL;
MEA	peptone 1g, glucose 20g, malt paste 20g, agar 20g, distilled water 1000mL;
CYA	K_2HPO_4 1g, Char's mother liquor 10 mL, yeast infusion powder 5g, sucrose 30g, agar 15g, distilled water 1000 ml.

Figure S1. ^1H NMR (600MHz, $CDCl_3$) spectrum of compound **1**;

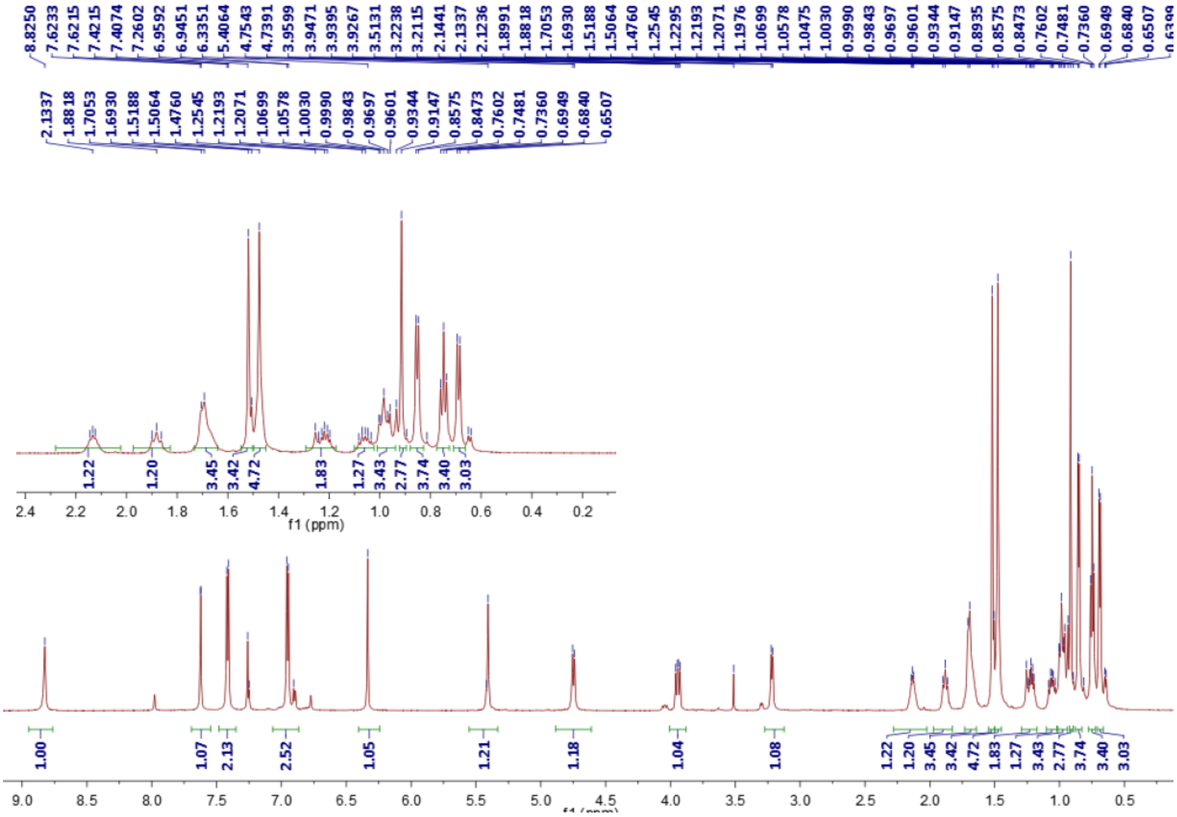

Figure S2. ^{13}C NMR (125 MHz, $CDCl_3$) and DEPT spectra of compound **1**;

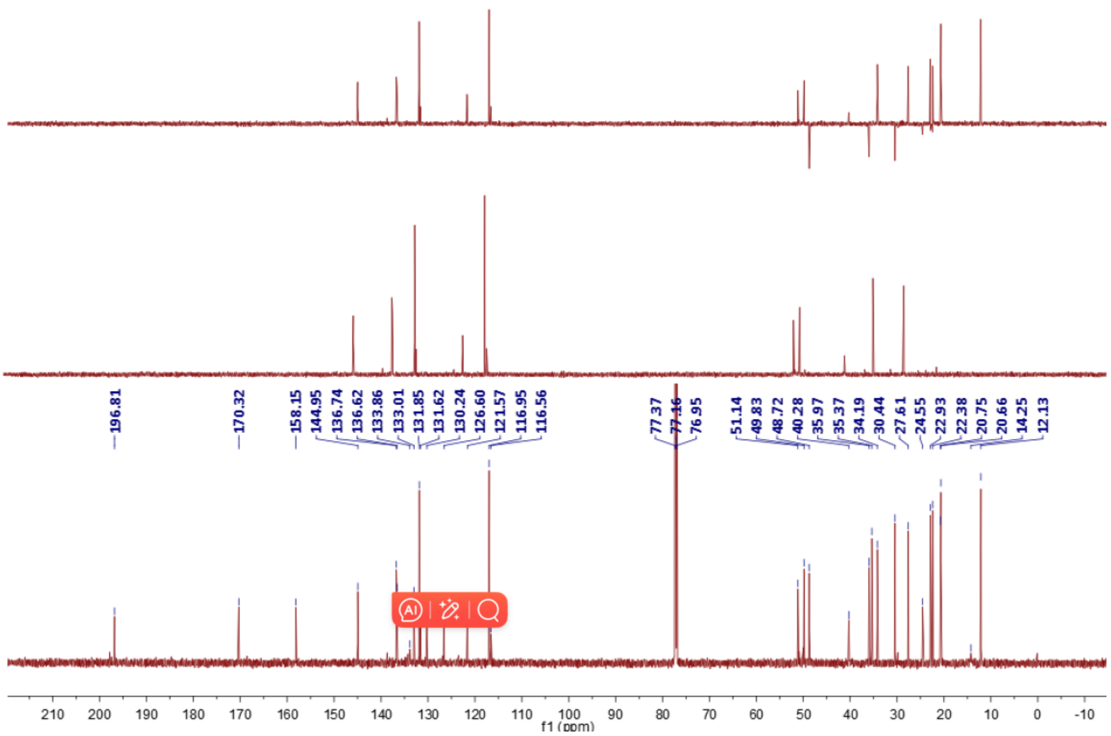

Figure S3. COSY spectrum of compound **1**;

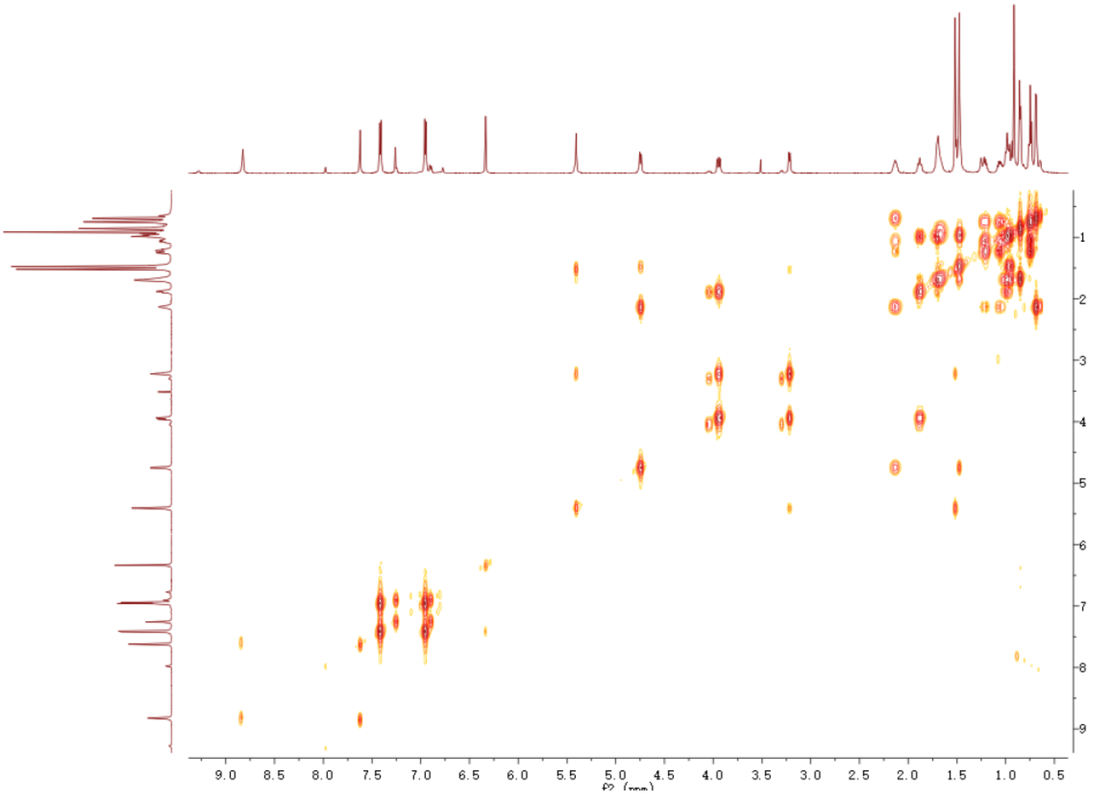

Figure S4. HSQC spectrum of compound **1**;

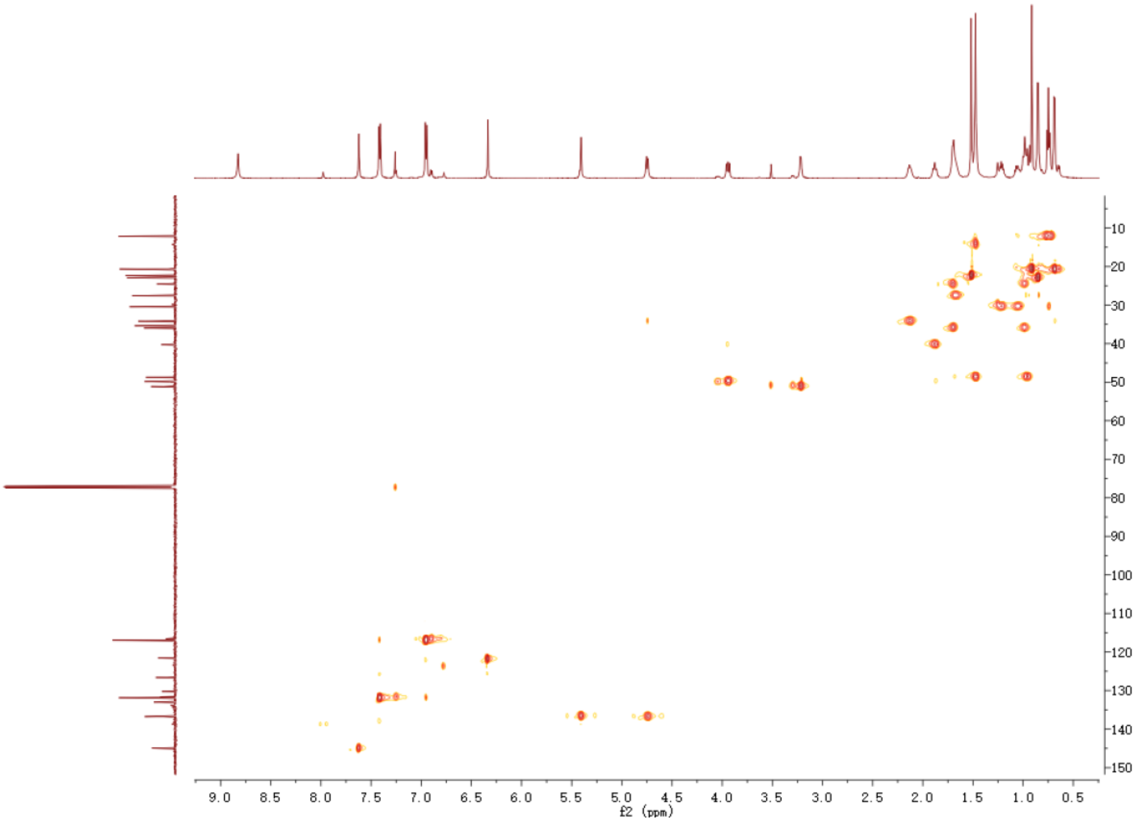

Figure S5. HMBC spectrum of compound **1**;

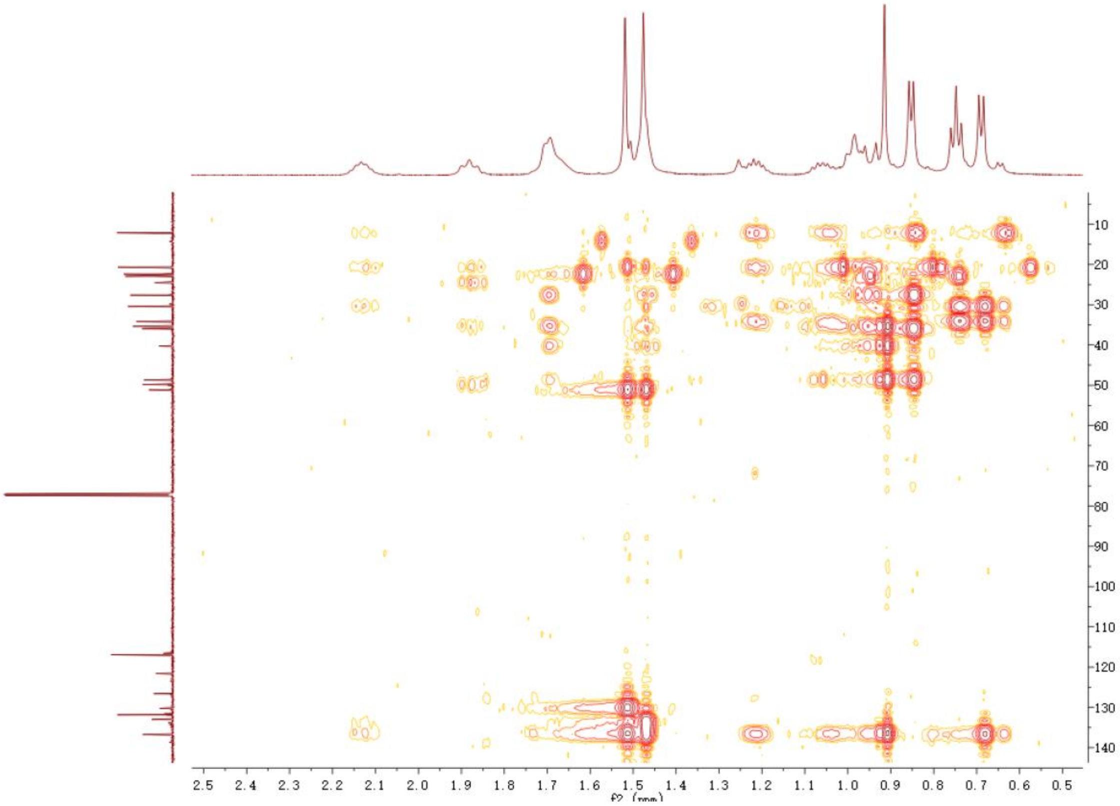

Figure S6. ^{1}H NMR (300 MHz, $CDCl_3$) spectrum of compound **2**;

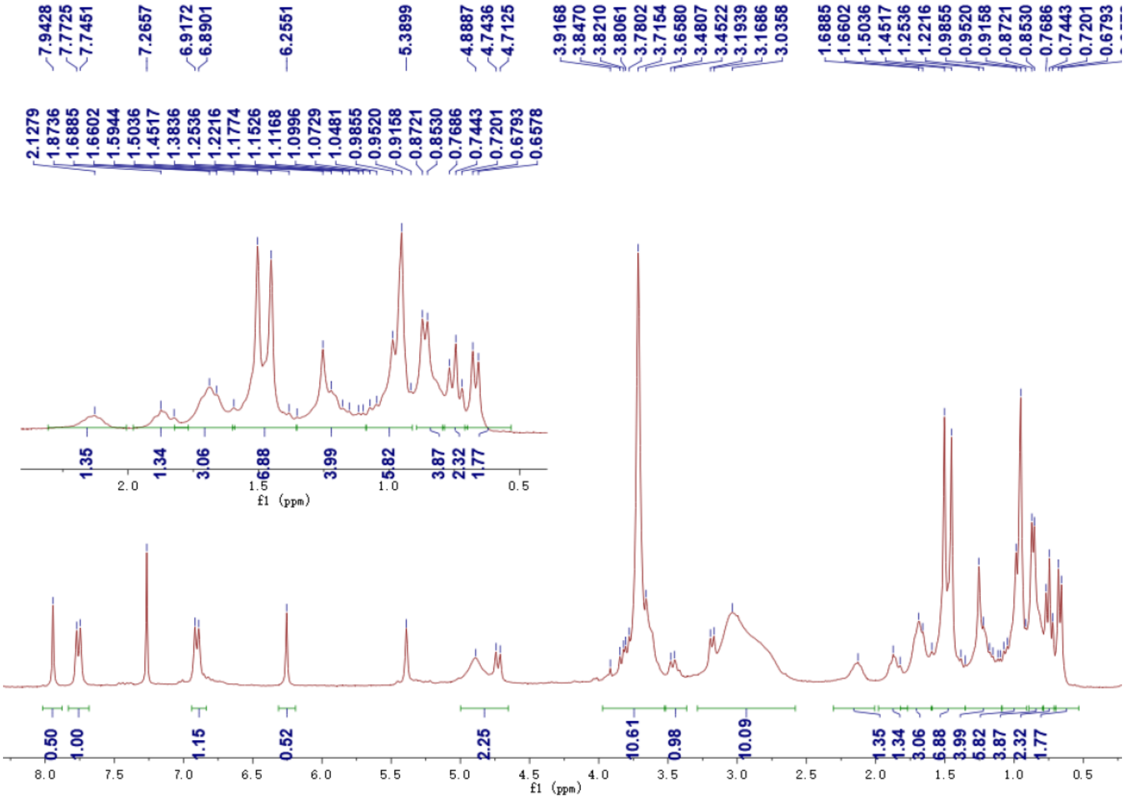

Figure S7. ^{1}H NMR (300 MHz, $CDCl_3$) spectrum of compound **3**;

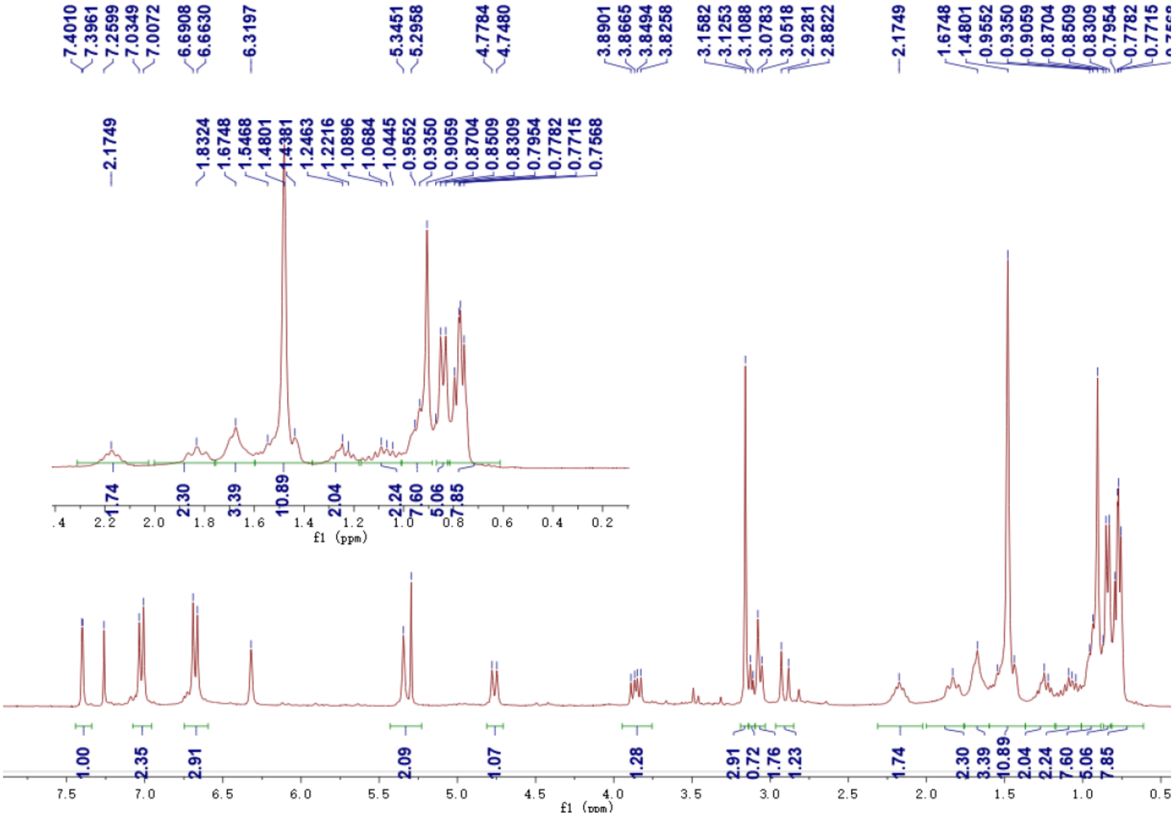

Supplement: Supplementary file 1 [file Data_Sheet_1.pdf]
